# Motor Coordination Disorders Evaluated through the Grid Test and Changes in the Nigral *Nrf2* mRNA Expression in Rats with Pedunculopontine Lesion

**DOI:** 10.3390/bs10100156

**Published:** 2020-10-13

**Authors:** Lisette Blanco-Lezcano, Esteban Alberti Amador, María Elena González Fraguela, Guadalupe Zaldívar Lelo de Larrea, Rosa Martha Pérez Serrano, Nadia Angélica Jiménez Luna, Dianet Camejo Rodríguez, Teresa Serrano Sánchez, Liliana Francis Turner, Bárbara Estupiñán Díaz, Yamilé Vega Hurtado, Isabel Fernández Jiménez

**Affiliations:** 1 Experimental Neurophysiology Department, International Center of Neurological Restoration (CIREN) Ave. 25 No. 15805 e/158 and 160, Playa, Havana 10300, Cuba; alberti@neuro.ciren.cu (E.A.A.); marie@neuro.ciren.cu (M.E.G.F.); dianetcr7@gmail.com (D.C.R.); teresa.serrano@infomed.sld.cu (T.S.S.); baby@neuro.ciren.cu (B.E.D.); yvega@neuro.ciren.cu (Y.V.H.); ifernandez@neuro.ciren.cu (I.F.J.); 2 Faculty of Medicine, Autonomous University of Queretaro, Clavel Street No. 200, Col. Prados de la Capilla, Santiago de Querétaro, Querétaro 76176, Mexico; apizl@yahoo.com.mx (G.Z.L.d.L.); rositaperezserrano@gmail.com (R.M.P.S.); nadia.jimenez@uaq.mx (N.A.J.L.); 3 Experimental Group: “Experimental Models for Zoo-Human Sciences”, Faculty of Sciences, Tolima University, 42nd Street, Barrio Santa Elena, Parte Alta CP 730001, Colombia; lilycolcuba@gmail.com

**Keywords:** pedunculopontine nucleus, grid test, *Nrf2*, neurodegeneration

## Abstract

Neurotoxic lesion of the pedunculopontine nucleus (PPN) is known to cause subtle motor dysfunctions. However, motor coordination during advance on a discontinuous and elevated surface has not been studied. It is also not known whether there are changes in the mRNA expression of nuclear factor (erythroid-derived 2)-like 2 (*Nrf2*) in nigral tissue. Methods: The effects of the unilateral neurotoxic lesion of the PPN in motor coordination evaluated through grid test and *Nrf2* mRNA expression in nigral tissue were evaluated. Two experimental designs (ED) were organized: ED#1 behavioral study (7 and 30 days after PPN lesion) and ED#2 molecular biology study (24 h, 48 h and 7 days) after PPN lesion. Results: ED#1—The number of faults made with left limbs, were significant higher in the lesioned groups (*p* < 0.01) both 7 and 30 days post-lesion. The number of failures made by the right limbs, was also significantly higher (*p* < 0.05) vs. control groups. ED#2—*Nrf2* mRNA expression showed an increase 24 h after PPN injury (*p* < 0.01), followed by a peak of expression 48 h post injury (*p* < 0.001). Conclusions: Disorders of motor coordination associated with PPN injury are bilateral. The increased *Nrf2* mRNA expression could represent an adaptive response to oxidative stress in the nigral tissue following pontine injury.

## 1. Introduction

Starting from the knowledge that there is neurodegeneration in extranigral nuclei as part of the degenerative course of Parkinson’s disease (PD) [1,2], new perspectives have opened up to the researcher focused on the selective or directed lesion of these nuclei and their relationships with nigral degeneration [3]. In this sense, the pedunculopontine nucleus (PPN) and substantia nigra pars compacta (SNpc) shares common physiologic properties among them, such as that both nuclei have highly branched axons, develop broad spikes and are calcium-dependent autonomous pacemakers [4,5]. At the same time, PPN has been identified as one of the nuclei where the Lewy Bodies are present very early in the degenerative course of the disease [6].

The literature indicates that nigral electrical activity is modulated by pontine-nigral cholinergic signals mediated by nicotinic and muscarinic receptors, which are expressed in nigral dopaminergic cells. [7]. It has also been pointed out that the modification of cholinergic synapses can increase the vulnerability of nigral dopaminergic neurons to oxidative events that activate cell death programs [8].

Our group has published evidence supporting the concurrence of events that increase the risk to nigral degeneration together with molecular and biochemical changes, suggesting compensatory mechanisms at nigral level in rats with a neurotoxic lesion of PPN [9,10,11,12]. In this sense, our results showed the occurrence of oxidative stress events at the nigrostriatal level, concomitant with a decrease in the nigrostriatal mRNA expression of vesicular monoamine transporters (VMAT2) and the dopamine transporter (DAT) in PPN-lesioned rats [9]. In connection with the compensatory mechanisms, our finding demonstrated an increase in the mRNA expression of brain-derived neurotrophic factor (BDNF) and tyrosine hydroxilase (TH), together a slight increase in gluthatione (GSH) level in nigral tissue of rats with PPN injury [10,11].

Finally, our group also showed that PPN neurotoxic injury induces changes in the nigral gene expression of transcription factors, such as Nurr 1 and Pitx3, which participate in the maintenance of the phenotype and dopaminergic homeostasis in the adult brain [12].

This group of evidence suggests the possibility that the nigral expression of other transcription factors could also be modified following the unilateral neurotoxic injury of PPN. In this sense, nuclear factor (erythroid-derived 2)-like 2 (*Nrf2*) is a transcription factor that regulates both the basal expression of genes under homeostatic conditions and the inducible expression of genes in a redox disorder scenario, which directly involves it in the molecular mechanisms that face oxidative stress [13]. *Nrf2* belongs to the Cap ‘n’ Collar (Cnc)-bZIP (basic leucine zipper) family of transcription factors [14]. In healthy conditions, *Nrf2* is sequestered in cytoplasm by Kelch-like ECH-associated protein1 (Keap1) [15,16,17]. However, under stressful conditions, the disruption of the Keap1-*Nrf2* complex occurs [15]. *Nrf2* translocates to the nucleus where it activates the antioxidant response element (ARE) and increases the transcription of *Nrf2*-regulated genes, many of them cytoprotective [18].

On the other hand, we and others have previously demonstrated that PPN neurotoxic lesion induces subtle motor deficits that can increase as the difficulty of the motor task increases. Multiple behavioral paradigms have been used to assess motor disability in rats with PPN injury, including: the staircase test [19], the vermicelli test [20], the bar and the cylinder test [9] and the footprint test [10,21].

In this work, we propose the study of the very early (seven days) and later (30 days) effects of the PPN lesion on the rat’s motor coordination through the grid test. The grid test involves a challenge for the neural mechanisms that guarantee optimal grip, balance, motor coordination and ambulation on an elevated and discontinuous surface [22]. This test has been used previously in the evaluation of disorders motors of mice treated with MPTP [23,24].

The results exposed in this work represent a continuation of the finding published by our group, which points out that the PPN neurotoxic injury increases the risk of nigral dopaminergic neurons to neurodegeneration. Our main objectives in the present work were the study of the effect of PPN neurotoxic injury on the motor coordination in two temporal windows (7 and 30 days after PPN lesion) and the early expression of other transcription factor in nigral tissue—in this case, *Nrf2*.

## 2. Materials and Methods

### 2.1. Experimental Subjects

Male Wistar rats weighing 200–350 g, from the Center for Breeding of Laboratory Animals (CENPALAB), Mayabeque, Cuba, were housed 5 per cage under a temperature of 22–24 °C, with a relative humidity of 60% ± 5% and and cycles of 12 h of light and 12 h of darkness. Water and food were provided ad libitum. Experiments were carried out in accordance with the Cuban Regulations for the Use of Laboratory Animals (CENPALAB 1997). Research Ethics Committee of CIREN analyzed and approved the protocol in September 2019 (Code: ET.7-2019(3)).

### 2.2. Surgical Procedure

**Figure 1:**
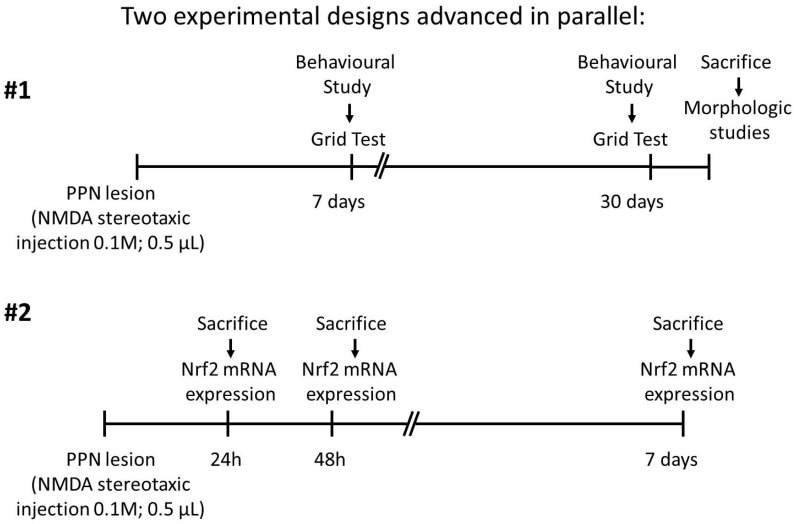
Two experimental designs progressed in parallel: #1. Behavioral study; #2. Molecular biology study. Unilateral neurotoxic lesion of right pedunculopontine nucleus was performed by injection of N-methyl-D-aspartate (NMDA) (Sigma, St. Louis, MO, USA) solution at a rate of 0.1 µL/min using a 5 µL Hamilton syringe. Experimental design #1: Motor coordination was studied by means of Grid test 7 and 30 days after pedunculopontine nucleus injury. When the behavioral studies concluded, the rats were sacrificed for morphological studies. Experimental design #2: Nigral *Nrf2* mRNA expression was studied 24 h, 48 h and 7 days after PPN injury. The selection of lesioned/sham-operated rats to form the 48-h and 7-days post injury group was completely randomized.

The rats were anesthetized with chloral hydrate (480 mg/kg intraperitoneal; i.p.) and placed in a frame of stereotactic surgery (David Kopf Instruments, Tujunga, CA, USA). The details of the procedure followed in the surgery have already been published previously by our group [9,10]. In brief, the neurotoxic lesion of the PPN was performed by means of the injection of an N-methyl-D-aspartate (NMDA) (Sigma, St. Louis, MO, USA) solution in the coordinates (mm) corresponding to the PPN, according to the Paxinos and Watson atlas (2007) [25] AP: −7.80 ML: 1.60 DV: −7.60 (Figure 1). At the end of the surgical procedure, the rats remained in postoperative care until their complete recovery. Sham lesion of the PPN (sham-operated group): the surgical procedure was the same as that followed for injection of the neurotoxin but in place of the neurotoxin the rats received 0.5 μL of physiological saline solution.

The rats were injured and assigned randomly to one of the two experimental designs that progressed in parallel: behavioral study and molecular biology study. The details of each are shown in Figure 1.

### 2.3. Evaluation of Motor Coordination

Motor coordination was studied seven and 30 days after PPN neurotoxic injury, through the grid test [22]. Behavioral studies were conducted under appropriate conditions of silence and lighting. Prior to the start of each trial, the rats were gently restrained and the right fore and hind limbs were stained with harmless ink so that they were easily identifiable in the images obtained during the study.

### 2.4. Experimental Set

**Figure 2:**
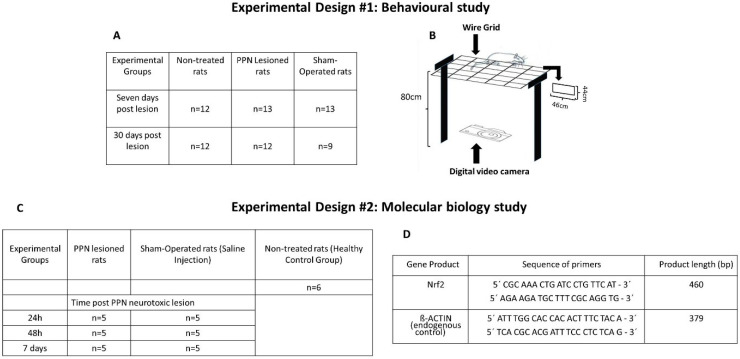
Main details of each experimental design (number of rats in each experimental group, set details, etc.). (**A**) Number of samples of each experimental group participating in the behavioral study. (**B**) Diagram of the grid apparatus and experimental set. Note the rectangular shape (46 × 44 cm) of the grid holes and the placement of a digital video camera that allows the moving image of the rat to be taken from a ventral position. (**C**) Number of samples of each experimental group participating in the molecular biology study. (**D**) Sequence of primers for RT-PCR.

Rats were placed on an elevated (height = 80 cm) wire grid (46 × 44 cm), with 5.5 × 3.5 cm rectangular holes and allowed to move freely for 60 sec while being videotaped from below with a digital video camera, as previously described [26] (Figure 2A,B). 

The following variables were determined from the analysis of the images obtained during the behavioral evaluation:

(a) Total number of failures committed by the limbs on each side,
(b) Number of failures made with each limb individually,
(c) Time delayed to the first failure.

Failure was considered when one of the limbs was not correctly positioned and the rat’s grip was compromised, or the limb was suspended inside one of the holes in the metal grid.

Taking into account that the body weight has a notable influence on the tests that evaluate motor coordination and balance [27], at the end of each behavioral evaluation session, the body weight of the experimental subjects was recorded.

The groups of rats in which motor coordination was evaluated 7 and 30 days after PPN injury were paired with their corresponding weight control groups.

A summary of the experimental groups involved in the behavioral study is shown in Figure 2A.

### 2.5. Molecular Biology Studies

Sample collection: Our group has already published the details of this procedure previously [12]. In brief, the dissections of the tissues were carried out under stereoscopic microscope (Wild Heerbrugg Stereo Microscopes, Heerbrugg, Switzerland). The animals were sacrificed by decapitation, after being anesthetized with chloral hydrate (480 mg/kg i.p.). The first step was to separate both hemispheres. Next, in the ventral portion of the right hemisphere, the mesencephalon was divided with a cut at the caudal border of median eminence perpendicular to the long axis of mesencephalon [28]. The substantia nigra-rich tissue was isolated by cutting at a third of the way from the dorsal midbrain with a blade slanted approximately at a 45° angle toward the researcher, as previously described [29]. Although we attempted to dissect the compact part of the SNpc only, a minor portion of the adjacent tissue could also be included to the final sample. The tissues were always dissected by the same investigator and under the same conditions. The molecular studies were performed on the right nigrostriatal tissue, ipsilateral to the PPN lesion. All of the brain tissues were stored at −80 °C until analysis.

### 2.6. RT-PCR Analysis for Gene Expression of Nrf2

Transcription factors mRNA expression was studied at 24 h, 48 h, and 7 days after the PPN neurotoxic lesion. The total RNA was extracted from the nigral tissue of 40 rats using TRIzol^®^ Reagent (Invitrogen, Carlsbad, CA, USA). The samples were eluted in 30 µL de UltraPureTM Distiled Water (Invitrogen, Waltham, Massachusetts, USA) and quantified in the in the NanoDrop OneC spectrophotometer (THERMO Scientific, Waltham, Massachusetts, USA) at 260 nm. Samples had 260–280 ratios in the range 1.8–2.1 they were selected, and their integrity was determined by 1% agarose gel electrophoresis. The RNA concentrations were registered, and the samples were stored at −70 °C. To synthesize cDNA, 1 µg of total RNA was reverse transcribed with ImProm-IITM Reverse Transcription System following the manufacturing protocol without modifications (PROMEGA, USA). The PCR was performed at SelectCycler II (Select BioProducts, Wembley, Western Australia, Australia) using 3 µL of cDNA mixed with 2 mM dNTPs, 6 mM MgCl_2_, 0.4 µM for each primer and 1 U of Taq DNA polymerase (Radiant^TM^, Alkali Scientific Inc, Fort Lauderdale, Florida, USA). Cycling conditions were as follows: 95 °C for 1 min; 40 cycles of 95 °C for 15 s; 64 °C (annealing temperature) for 1 min; 72 °C for 1 min; one last step of 72 °C for 10 min. Figure 2D shows the data concerning the sequence of each primer used for RT-PCR and the size of each gene product. 

PCR products were separated by 2% agarose/ethidium bromide gel electrophoresis and visualized under UV light. Each electrophoresis was performed twice, followed by a semi-quantification analysis. For the semi-quantitative study, the free online program ImageJ (Version 1.44; Wayne Rasband, National Institute of Health, Bethesda, MD, USA; http://imagej.nih.gov/ij) was employed. The analysis was performed according to the published method [30]. The background activity of the target band was subtracted and then normalized using β-actin as a reference.

### 2.7. Morphological Studies

The morphological studies were performed in 5 rats of each experimental group. Rats were anesthetized (chloral hydrate, 420 mg/kg body weight, i.p.) and perfused transcardially with physiological saline solution followed by 4% formaldehyde. The brains were removed and blocked in the coronal plane and paraffin-processed.

The coronal sections (thickness = 10 µm) were taken from the anterior cerebellum through to anterior substantia nigra and placed on gelatin-coated slides. Every section was heated, deparaffinized and rehydrated. Two series corresponding to PPN coordinates were stained according to Cressyl Violet protocol in order to locate the correct site of neurotoxin injection. 

### 2.8. Immunohistochemistry

For the immunohistochemical study, a complete series constituted by twelve coronal sections of substantia nigra from each rat brain was used. After washing in PBS, sections were incubated for 20 min in 3% H_2_O_2_–PBS. They were then permeabilized with 0.1% Tween-PBS and treated with 3% BSA-0.1% Tween-PBS for 45 min. Thereafter, they were incubated overnight with the primary antibody (anti-neuronal nuclear protein (NeuN), Chemicon, Merck Millipore 1:1000) diluted in 1% BSA–PBS. NeuN was detected by incubation for 1.5 h with a biotin-conjugated rabbit anti-mouse antibody (Sigma, St. Louis, Missouri, 1:500) and revealed after 2 h of exposition with avidin–biotin peroxidase ABC kit (Vectastain, Vector Labs, CA, USA) and 3, 30-diaminobenzidine (DAB; Peroxidase Substrate Kit; Vector Labs).

The neuron counts were performed in the sections corresponding to right SN from PPN lesioned rats (7 and 30 days) and Sham-operated groups. The images were obtained by means of a high-resolution digital camera (DP 71) coupled to a conventional optical microscope (Olympus BX51, Tokyo, Japan). Cell numbers were counted following a systematically random sampling scheme. Neuronal characteristics included a neuronal morphology with a clearly defined nucleus. The counting was performed manually using the cell counter plugin of the free online program ImageJ (Version 1.44; Wayne Rasband, National Institute of Health; http://imagej.nih.gov/ij). Neuronal density (number of cells/mm^2^) was estimated by dividing the number of cells between the areas of a complete field of the 40× objective (0.615 mm^2^).

Finally, in order to analyze if the nigral neurons modify their surface area following the PPN injury, images of 20 NeuN-stained cell bodies of the SNc from the sections where the number neurons were previously determined, were captured using a camera mounted to the microscope under 40× objective. The cell body contours were traced and their surfaces were measured using Image J software (Version 1.44; Wayne Rasband, National Institute of Health; http://imagej.nih.gov/ij). The data were exported to Excel software and finally were analyzed using Statistica 8.0 software.

The cell count and the determination of the surface area were carried out by researchers who were blind to the experimental group under study.

### 2.9. Statistical Analysis

Normal distribution and homogeneity of variance of the data were analyzed applying the Kolmogorov–Smirnov and Levene tests, respectively. The comparison between experimental groups of the variables—rats weight, time delayed to the first failure and *Nrf2* mRNA expression—was carried out by a one-way analysis of variance (ANOVA) followed by a Tukey Test. For these variables, the values are expressed as mean ± SEM.

On the other hand, the variables—total number of failures committed by the limbs on each side, number of failures made with each limb individually, neuronal density and surface area—did not show normal distribution. These variables were compared between experimental groups by means of non-parametric test Kruskal–Wallis followed by a Newman–Kewls test. The variables corresponding to behavioral study are presented as median of the sample. The variables equivalent to the morphological study are presented as mean ± SEM.

For all analyses, a significance level of 0.05 was considered using the Statistica 8.0 software (StatSoft Ink, Tulsa, OK, USA) software. The graphics were elaborated using GraphPad Prism 5.0 software (GraphPad Software, California, EEUU).

## 3. Results

In the Results and Discussion sections, the limbs on the left side, contralateral to the lesion, will be referred to as contralateral. Meanwhile, the limbs on the right side, ipsilateral to the lesion, will be referred to as ipsilateral.

### 3.1. Evaluation of Motor Coordination through Grid Test 

**Figure 3:**
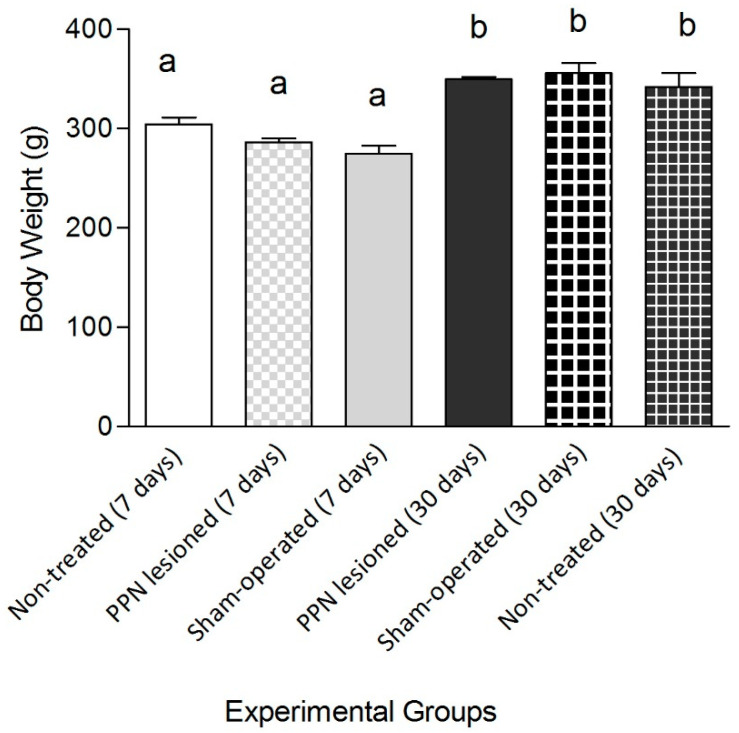
Comparative study of the body weight between experimental groups. The analysis of the body weight showed significant differences between experimental groups (F_(5, 65)_ = 17.11 *p* ≤ 0.001). The body weight segregated the groups where the behavioral study was carried out seven and 30 days after pedunculopontine nucleus lesion. One-way ANOVA test. a vs. b: *p* < 0.01. The bars represent X ± E.E.M.

The variable body weight showed a significant increase (F_(5, 65)_ = 17.11 *p* ≤ 0.001) in the groups evaluated 30 days after PPN neurotoxic lesion (Figure 3). Taking into consideration that the body weight is a very important variable for the balance and motor coordination analysis, the following comparisons were carried out in the groups with 7 and 30 days post lesion separately.

**Figure 4:**
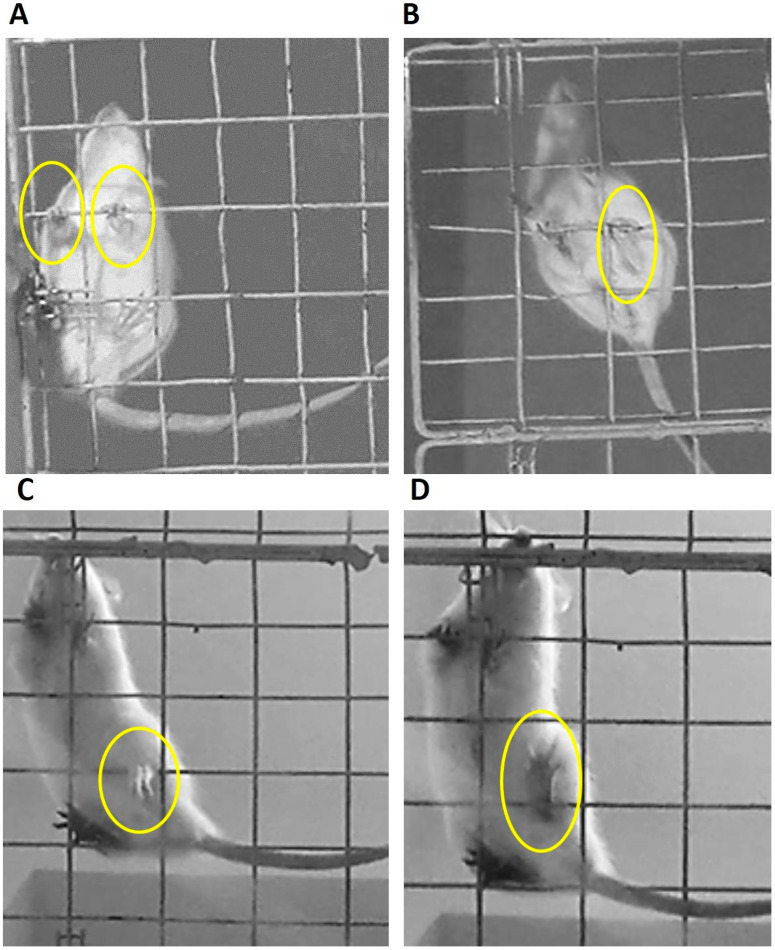
Clamping patterns adopted by the rats during its advance over grid metal. (**A**,**B**) Corresponding to control groups (non-treated and sham operated rats). (**A**) Correct positioning of the fingers around the grid in the fore limbs. (**B**) Grip of the hind limbs with adequate support and flexion of the fingers. (**C**) Left hind limb of the lesioned rat suspended from the hole of the grid showing an imprecise grip. The partial support is achieved with the external distal edge of the limb. (**D**) Left hind limb of the lesioned rat presents sustained opening of the digits and their hyperextension.

From the qualitative point of view, the analysis of the images recorded during behavioral studies throws into relief subtle differences in the locomotion of the experimental subjects in a discontinues and elevated support superficies. Control rats keep the digits flexed around the crossbars allowing them optimal grip (Figure 4A,B). Meanwhile, the lesioned rats, for both post injury times studied (7 and 30 days) show a clamping pattern characterized by the sustained opening of the digits and their hyperextension in both left limbs (forelimb and hind limb). In addition, the injured rats presented a partial support of the left hind limb. The support is achieved with the external distal edge of the limb during the advancement on the metal grid (Figure 4C,D).

#### 3.1.1. Motor Coordination Seven Days after PPN Lesion

The total number of failures made by the ipsi and contralateral limbs (without discriminating between the forelimb and the hind limb) was evaluated as a first approach to the unilateral disability that the experimental subjects presented seven days after PPN injury.

**Figure 5:**
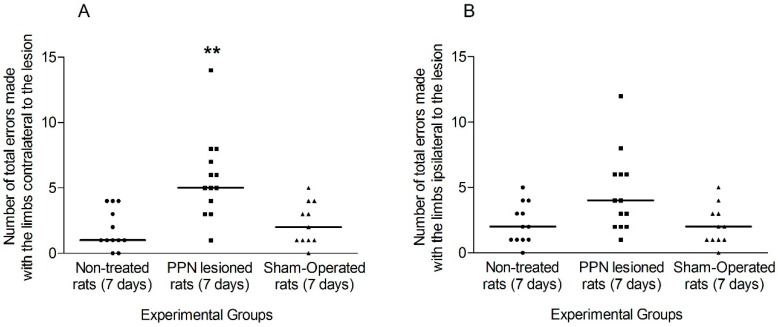
Comparison of the total faults made by the limbs of each side during the execution of Grid Test seven days after pedunculopontine nucleus lesion. (**A**) Faults exhibited by the contralateral limbs (H_(2, 38)_ = 12.34 *p* < 0.01). (**B**) Faults corresponding to ipsilateral limbs (H_(2,38)_ = 5.00 *p* > 0.05). Kruskal–Wallis test. ** *p* < 0.01. The points represent the number of errors of each experimental subject. The horizontal lines represent the median of each sample.

The faults in the contralateral limbs revealed a significant increase in the lesioned rats (H_(2, 38)_ = 12.34 *p* < 0.01). In the case of the ipsilateral limbs, although a tendency to increase in this variable was observed in the group of lesioned rats, this tendency did not reach statistical significance (H_(2,38)_ = 5.00 *p* > 0.05) (Figure 5A,B).

The comparison of the faults of each limb separately between experimental groups allowed for evaluating the individual contribution of each limb to the total number of the faults of each side.

**Figure 6:**
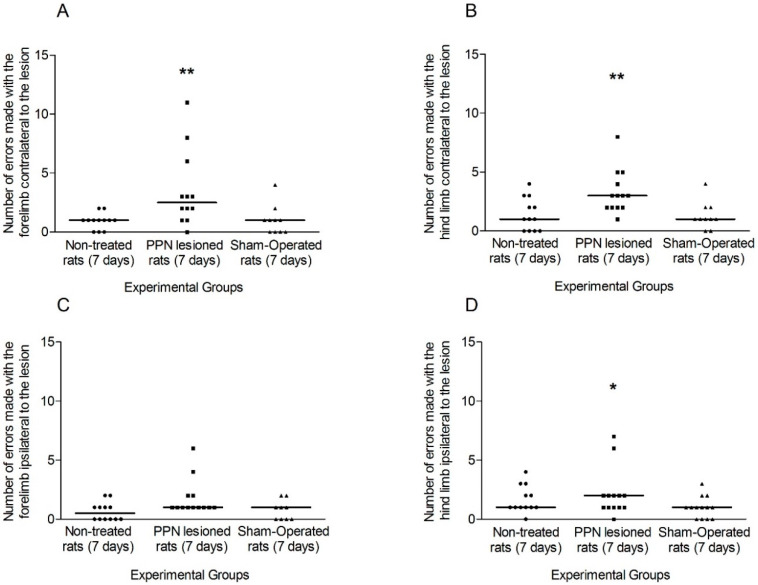
Comparison of the faults made by each limb separately during the execution of Grid Test seven days after pedunculopontine nucleus lesion. (**A**) Faults made by the contralateral forelimb (H_(2,38)_ = 10.91 *p* < 0.01). (**B**) Faults corresponding to contralateral hind limb (H_(2,38)_ = 6.71 *p* < 0.05). (**C**) Faults made by the ipsilateral forelimb (H_(2, 38)_ = 3.81 *p* > 0.05) (**D**) Faults corresponding to ipsilateral hind limb (H_(2, 38)_ = 8.45 *p* < 0.05). Kruskal–Wallis test. * *p* < 0.05, ** *p* < 0.01. The points represent the number of errors of each experimental subject. The horizontal lines represent the medians of each sample.

The number of faults made by the contralateral fore and hind limbs showed a significant increase (H_(2,38)_ =10.91 *p* < 0.01; H_(2,38)_ = 6.71 *p* < 0.01, respectively) in the PPN lesioned rats (Figure 6A,B). In the case of ipsilateral forelimb, the number of faults was not significant between experimental groups (H_(2, 38)_ = 3.81 *p* > 0.05), while this variable presented a significant increase for the ipsilateral hind limb (H_(2, 38)_ = 8.45 *p* < 0.05) in the PPN lesioned rats (Figure 6C,D).

The time delayed for the rats before the first fault, although showing a tendency to decrease in the group of PPN lesioned rats, was not significant (F_(2, 35)_ = 2.10 *p* > 0.05) (Table 1).

#### 3.1.2. Motor Coordination 30 Days after PPN Lesion

The analysis of the fault 30 days after the lesion, it followed the same outline of the analysis carried out for the seven days after the lesion.

**Figure 7:**
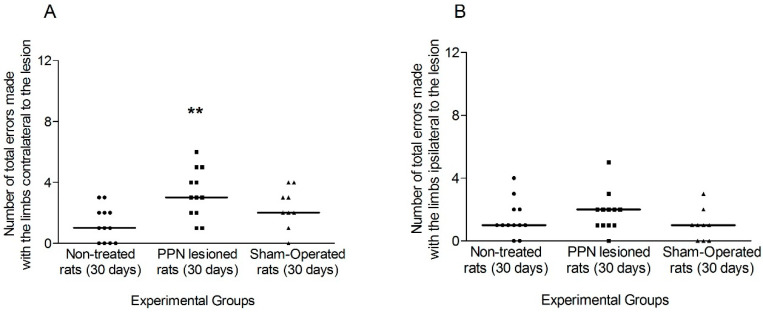
Comparison of the total faults made by the limbs of each side during the execution of Grid Test 30 days after pedunculopontine nucleus lesion. (**A**) Faults made by the contralateral rats’ limbs (H_(2,33)_ = 11.87 *p* < 0.01). (**B**) Faults corresponding to ipsilateral limbs (H_(2,33)_ = 5.05 *p* > 0.05). Kruskal–Wallis test. ** *p* < 0.01. The points represent the number of errors of each experimental subject. The horizontal lines represent the median of each sample.

The faults in the contralateral limbs revealed a significant increase in the lesioned rats (H_(2,33)_ = 11.87 *p* < 0.01). In the case of ipsilateral limbs, also in the analysis of the 30 days after the lesion, although a tendency to increase in the faults was observed in the lesioned rats, this tendency did not reach statistical significance (H_(2,33)_ = 5.05 *p* > 0.05) (Figure 7A,B).

**Figure 8:**
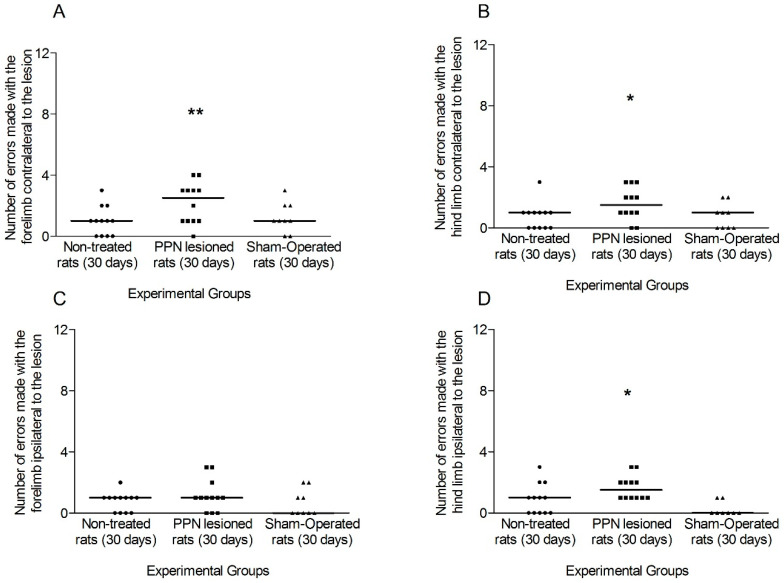
Comparison of the faults made by each limb separately during the execution of grid test, 30 days after pedunculopontine nucleus lesion. (**A**) Faults made by the contralateral forelimb (H_(2,33)_ = 9.65 *p* < 0.01). (**B**) Faults corresponding to contralateral hind limb (H_(2,33)_ = 8.03 *p* < 0.05). (**C**) Faults made by the ipsilateral fore limb (H_(2,33)_ = 1.42 *p* > 0.05) (**D**) Faults corresponding to ipsilateral hind limb (H_(2,33)_ = 4.90 *p* < 0.05). Kruskal–Wallis test. * *p* < 0.05, ** *p* < 0.01. The points represent the number of errors of each experimental subject. The horizontal lines represent the median of each sample.

In connection to the individual contribution of each limb, the comparison of the faults made by the contralateral, fore and hind, limbs between experimental groups revealed a significant increase for the lesioned rats (forelimb H_(2,33)_ = 9.65 *p* < 0.01; hindlimb H_(2,33)_ = 8.03 *p* < 0.05)) (Figure 8A,B). For the ipsilateral limbs, the comparison of the forelimb faults was not significant between experimental groups (H_(2,33)_= 1.42 *p* > 0.05) meanwhile the hindlimb faults revealed a slight significant increase (H_(2,33)_= 4.90 *p* < 0.05) for the 30 days injured rats in comparison to the control groups (Figure 8C,D).

Again, the comparison of the time delayed to the first fault showed a tendency to decrease in the 30 days lesioned rats, but this tendency did not reach significant differences (F_(2,30)_ = 1.70 *p* > 0.05) (Table 2).

### 3.2. Molecular Biology Results 

**Figure 9:**
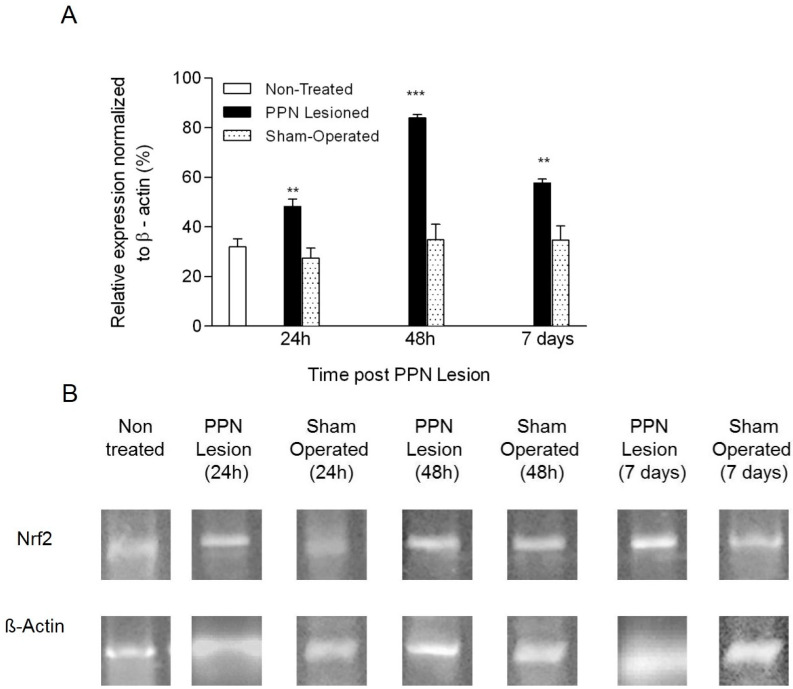
Effect of pedunculopontine nucleus (PPN) neurotoxic lesion on the nuclear factor (erythroid-derived 2)-like 2 (*Nrf2*) mRNA expression in nigral tissue. (**A**) Comparison between experimental groups 24 h, 48 h and 7 days after PPN lesion (F_(6,29)_ = 39.08 *p* < 0.001). The first bar (white bar) represents the non-operated group. Asterisks correspond to statistical differences between PPN lesion and both control groups (non-operated and sham operated). (**B**) Agarose/ethidium bromide gel electrophoresis bands representative of the semi-quantitative RT-PCR study for *Nrf2* mRNA expression. One-way ANOVA test. ** *p* < 0.01; *** *p* < 0.001.

*Nrf2* mRNA expression showed a significant increase in nigral tissue following the PPN neurotoxic lesion (F_(6, 29)_ = 39.085 *p* < 0.001). The behavior of this variable was characterized by an early significant increase 24 h after PPN injury (*p* < 0.01) followed by a peak of expression 48 h post injury (*p* < 0.001). In a later time window (7 days after PPN injury), *Nrf2* mRNA expression, although lower, remains significantly increased in relation to control groups (*p* < 0.01) (Figure 9A,B).

### 3.3. Morphological Results

**Figure 10:**
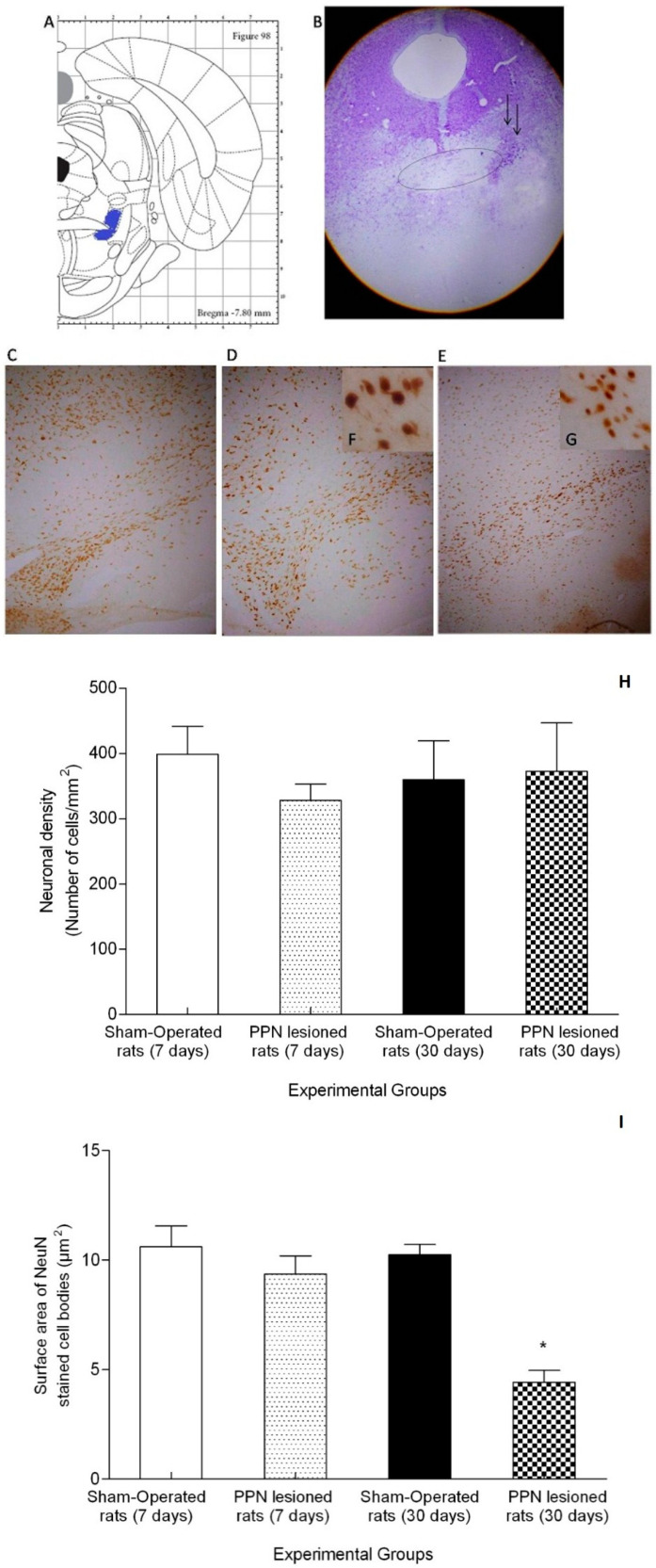
Morphological study. (**A**) Schematic diagrams of the sections showing pedunculopontine nucleus (PPN) area (blue color) adjacent to the superior cerebellar peduncle (SCP) in the rat brain adapted from Paxinos and Watson (2007). The diagram corresponds with the antero posterior coordinate used in the present study. (**B**) Representative photomicrographs of the cresyl violet study showing the location of the PPN lesion (4×). The ellipse represents the SCP bundle in whose distal portion the area corresponding to the pedunculopontine nucleus is located. Little black arrows indicate the lesion area. C-G Representative photomicrographs of the immunohistochemical study for anti-neuronal nuclear protein (NeuN) in nigral coronal sections. (**C**) Sham-operated rats (10×). (**D**) Right substantia nigra (seven days post PPN lesion) (10×). (**E**) Right substantia nigra (30 days post PPN lesion) (10×). (**F**,**G**) Magnification (40×) of the image (**D**,**E**). (**H**) Comparison of neuronal density between the right substantia nigra of PPN-lesioned rats (seven and 30 days after lesion) and Sham-operated rat groups (Z = 3.74 *p* > 0.05). (**I**) Comparison of surface area between the right substantia nigra of PPN-lesioned rats (seven and 30 days after lesion) and Sham-operated rat groups (Z = 9.77 *p* < 0.05). * *p* < 0.05.

The morphological study corroborated the results previously published by our group. The stereotactic injection of NMDA solution covered the full extent of the anterior–posterior plane of the PPN (approximately between the stereotactic coordinates AP −7.20 mm to AP −8.16 mm). The zone of injury was circumscribing to the distal part of the decussation of the superior cerebellar peduncle. According to the Cresyl Violet study, the lesion does not appear to compromise these fibers (Figure 10A,B).

The immunohistochemical study for NeuN revealed that the zone coincident with substantia nigra did not present neural depopulation either at 7 days or at 30 days following the PPN injury (Figure 10C–G). Comparison of neuronal density showed non-significant differences between the right SN from NMDA-lesioned rats (7 and 30 days) and Sham-operated groups (Z = 3.74 *p* > 0.05) (Figure 10H). Regarding to surface area, this variable showed a significant decrease in the neuronal cell bodies from SNpc following 30 days of PPN neurotoxic lesion (Z = 9.77 *p* < 0.05) (Figure 10I).

## 4. Discussion

From a behavioral point of view, the main results of this work demonstrate that the coordination and grip disorders associated with neurotoxic PPN injury in rats are bilateral and are installed early, seven days after the pontine injury. These motor dysfunctions remain unchanged until at least 30 days after the injury. At the same time, our results show that PPN neurotoxic injury is associated with a gradual increase in the nigral gene expression of *Nrf2*, which has not been previously reported in the literature. These results are concomitant with a reduction in the nigral neuronal surface area seven days after PPN neurotoxic lesion, although we did not observe a significant change in the neuronal density.

### 4.1. Neurotoxic PPN Injury Affects Motor Coordination during Ambulation over a Discontinuous and Elevated Horizontal Support Surface

Our results show that the unilateral neurotoxic lesion of PPN promotes early dysfunctions of the balance and motor coordination that are reflected in the significant increase in the number of faults made with fore and hind limbs, contralateral to PPN lesion. In connection with the temporary behavior, the evidence suggests that the dysfunctions of motor coordination evaluated with the grid test are installed very early after PPN lesion. Thirty days after the lesion, these dysfunctions do not modify in a remarkable way. The left limbs, contralateral to the lesion, continue being those that cause a larger number of faults during the motor task execution. However, it is valid to highlight that the number of faults made by the ipsilateral hind limb was also significantly superior in the group of injured rats at seven and the 30 days after PPN lesion. This result suggests that, although it is a model of unilateral lesion, we cannot ignore a bilateral component in the pontine lesion effects.

The execution in the grid test demands accurate limb placement and thus substantial motor control to cross this discontinuous surface without making faults that commit balance and postural stability [22,31]. The horizontal grid test is ideal for the assessment of skilled forepaw use in relation to distal musculature and digit manipulation [27]. In addition, this paradigm evaluates the sensorimotor coordination between fore and hind limbs, and it is able to measure the dysfunctions in the descendent motor control [32]. Consequently, this test has been used to evaluate the motor dysfunctions and the later recovery of these functions when some therapeutic strategies have been rehearsed in spinal cord lesion models, pyramidal tract lesion models, as well as in traumatic brain injury models (TBI) [22,33,34].

Other authors have used the grid test to evaluate subtle motor disorders of parkinsonian mice by exposition to moderate doses of MPTP [23,24]. To our knowledge, this is the first study that the grid test is applied to for studying the effects of the PPN neurotoxic lesion in motor coordination and digit manipulation. 

The analysis of the effects of neurotoxic injury of PPN on motor performance must take into account several essential aspects: (i) the multiple morpho–functional relationships of PPN with basal ganglia, the thalamus and the cerebral cortex [35]; (ii) the projections of the PPN to the spinal cord through the reticulospinal tract [35,36]).

The correct coordination between the fore and hind limbs requires optimal functioning of: (i) both pontine and bulbar reticulospinal tracts to start the gait cycle; (ii) corticospinal and rubrospinal tracts that control voluntary movements in rats [37]. Neurotoxic injury of PPN can compromise the processing of motor information by pontine neurons involved in the pontine reticulospinal tract, which runs through the anterior funiculus of the spinal cord and facilitates the activity of the extensor motor neurons [38]. The extensor muscles of the hip, knee, and ankle are essential in the gait pattern that distinguishes quadruped locomotion [39].

On the other hand, damage to the ascending pontine-thalamic projections, consistent with the PPN unilateral neurotoxic injury, may compromise the processing of sensory information that contributes to postural control [38].

Regarding the support strategy of the rats injured during their movement in the metal grid, the fact of keeping the digits separated and open reveals inadequacy and loss of effectiveness in the grip on the grid holes, as well as a poor control of muscle tone of the distal limbs. In a previous work, our group published that rats with unilateral PPN injury show an asymmetric behavior in the use of the forelimbs in a vertical exploration task [9]. In this sense, it was described that the limb contralateral to the lesion shows ineffective support to the wall of a transparent cylinder and explores it with some delay, compared to the ipsilateral limb (right) [9].

The pontine neurons, which are part of the mesopontine segment, behave as a very important interface between the basal ganglia and the brain stem [35]. In this modulating role, they participate in the control of postural and limb muscle tone during voluntary movements associated with locomotion [35]. Our results suggest that PPN neurotoxic injury compromises the integrative function of this nucleus in the control of muscle tone in both the axial and distal muscles. However, other studies could clarify more details of this aspect.

Another interesting detail is that the movement of lesioned rats on the grid does not accurately reproduce the mammalian quadruped walking pattern characterized by coordinated and alternate bilateral activation in the limb muscles [40]. The injured rats sometimes leap forward and made turns on the grid. The displacement always begins with the advance of the right forelimb ipsilateral to the lesion, which commands movement. Meanwhile, the left limbs combine the ineffective grip described above, along with a certain delay (very difficult to quantify) in the case of the hind limb. These observations, evaluated jointly, maintained the left side of the rat body with a delayed advance or subordinated the advance of the right side ipsilateral to the PPN injury.

The basic step pattern for most mammals is generated from spinal circuits that are under supraspinal control, mainly from the reticulospinal bundle and some contribution from both the corticospinal bundle and rubrospinal pathways that regulate the placement of the forelimbs [41]. The spinal motor neurons that control the forelimbs, which are located in the upper and lower thoracic and cervical spinal segments, are under the influence of the basal ganglia through their connections with the motor cortex and PPN, and the latter both with the reticulospinal pathway and with the spinal motor neurons directly [42]. It is possible that the supraspinal connections, in which the reticulospinal bundle intervenes, show different degrees of involvement following the PPN neurotoxic injury. This could explain the quantitatively different involvement of the anterior and posterior limbs ipsilateral to the injury.

On the other hand, the motor execution in the grid test involves an additional challenge, consisting in that the grid is located at a height of 80cm. Rats perform a visual height inspection for which they present their heads inside the openings (looking down) at certain intervals. This behavior has been described in other behavioral paradigms in which the support surface is located at a certain height, adding complexity to the execution of the motor task [9,43]. The literature indicates that visual height inspection can be consistent with the visuospatial and visuomotor processing necessary for the correct execution of the test [43]. 

In another sense, we consider it important to highlight the fact that the neurotoxic injury to the PPN also modifies the performance of the rat’s right hind limb. This result points to the fact that the pontine lesion has a bilateral scope. The literature suggests that PPN plays a determining role in the inter-hemispheric regulation of basal ganglia activity [44]. In the rat brain, the PPN is reciprocally connected to different ipsilateral basal ganglia nuclei and, at the same time, sends collateral branches to the thalamus, entopeduncular nucleus, ventral tegmental area, and contralateral SNpc [45]. These connections that cross to the opposite hemisphere, could sustain the bilateral effects of the lesion observed in the present work.

On the other hand, the rats evaluated seven days after the PPN injury show a remarkable tendency to make the first failure before the control rats. This behavior, far from reversing 30 days after the injury, continues with the same trend. This fact points to the fact that the processing of proprioceptive information that contributes to displacement and grip on a discontinuous elevated horizontal support surface, is very labile and, once damaged following the pontine injury early, is not recovered, at least not before the longest time window studied in the present work.

Although this variable did not reach statistical significance, we consider that its trend should be taken into account since it reinforces the criterion of dysfunction in motor coordination necessary for the execution of this behavioral paradigm. Considering the multiple morpho–functional relationships of the PPN with structures involved in motor control, from reference [35] we can hypothesize that the pontine injury causes the neural mechanisms that support postural control and ambulation to lose sensitivity and accuracy. This compromise might be more evident in the initial seconds after the experimental subject faces the challenge of wandering on the discontinuous elevated support surface.

### 4.2. Neurotoxic PPN Injury Modifies Nrf2 mRNA Expression in Nigral Tissue

The present study confirms that PPN neurotoxic injury triggers a slightly early (24 h after PPN lesion) increase in *Nrf2* nigral gene expression, reaching a peak of expression 48 h after pontine injury. The increase, although it was maintained during seven days after PPN lesion, at this time window, the expression level had already been significantly decreased in relation to 48 h after PPN lesion.

This temporal behavior may be part of a mosaic of molecular events that involves the nigral gene expression of other genes, such as: the *brain derived neurotrophic factor*, key in the survival of nigral dopaminergic neurons, *TH*, *VMAT* and *DAT*, essential in maintaining dopaminergic homeostasis, as well as *Nurr1* and *Pitx3*, those that play a role in maintaining the dopaminergic phenotype [10,11,12].

*Nrf2* is considered a master regulator of the antioxidant response [17]. Under physiological conditions, *Nrf2* is constitutively expressed and its expression is tightly regulated [46]. The literature suggests that under redox homeostasis, a repression of *Nrf2* activity occurs from its binding to the Keap 1 protein [47]. In this case, Nrf2 is ubiquitinated and subjected to proteasomal degradation [48]. Thus, Keap1 regulates *Nrf2* negatively by promoting its sequestration and degradation [48].

Keap1 is a cysteine-rich protein that anchors *Nrf2* to the cytoplasm of the cell, preventing its translocation to the nucleus and the transactivation of other genes and transcription factors [49]. However, evidence from knockout mice indicates that *Nrf2* shows subtle activity even under normal homeostatic unstressed conditions [13].

The literature indicates that the activation of *Nrf2* proceeds through the inactivation or inhibition of Keap1 [14]. The inactivation of Keap1 can be facilitated by a structurally diverse range of chemicals that possess the capacity to modify cysteine residues [13]. Quinones and hydroperoxides, together with another group of molecules, have been considered as a possible candidate for Keap1 inactivation due to interaction with the cysteine residues present in this protein [50].

Previous results from our laboratory indicate an increase in the nigral enzymatic activity of catalase, suggestive of high levels of H_2_O_2_ as early as 48 h after PPN lesion. This result followed on from an increase in nigral malondyaldehyde concentrations, indicative of lipid peroxidation events seven days after PPN lesion [9]. These findings demonstrate the gradual loss of nigral oxidative homeostasis following the PPN neurotoxic injury.

The moderate oxidative stress environment can facilitate the substrate for the reaction with Keap1 and posterior stabilization of *Nrf2*. This environment has been associated with increased activity of *Nrf2* both transcriptionally and translationally [51,52]. For the present experimental design, the moderate oxidative stress condition could be reach following 48 h after PPN lesion.

On the other hand, our group demonstrated an increase in the *TH* mRNA expression together with a decrease in the *VMAT* mRNA expression in nigral tissue, seven days after PPN lesion [11]. This combination was considered an early signal of alarm for the nigral dopaminergic neurons. Even if higher levels of dopamine were being synthesized, the dopamine vesicular storage would be compromised in this situation [11]. Vesicular packing prevents the cytosolic accumulation of DA and its subsequent oxidation to potentially neurotoxic molecules [53]. Therefore, we hypothesized that, in this condition, an increase in quinone production could be promoted as a result of the oxidation of dopamine not stored in vesicles. Quinones could maintain an *Nrf2* activation signal, which could explain that, although with lower levels, the *Nrf2* mRNA expression in nigral tissue still remains increased seven days after PPN neurotoxic injury. Additionally, in this time window, the highest concentrations of malondyaldehyde in the nigral tissue are presented [9], which could also contribute, to some extent, to the temporal behavior of the *Nrf2* mRNA expression observed in the present work. However, further studies will be necessary to confirm this hypothesis.

Experimentally, pharmacological strategies have been tested using the potentially activating role of the *Nrf2* pathway, shown by molecules such as quinones [54]. The literature indicates that astrocytes from adult and old rats are notably susceptible to MPP+ toxicity. This condition is reverted following pretreatment with the *Nrf2* activator tert-butyl-hydroquinone. Consequently, these experiments demonstrated an increase in the expression of antioxidant and cytoprotective enzymes [54].

The gradual increase (24–48 h after PPN lesion) in the nigral gene expression of *Nrf2* could support the transient increase in the nigral concentrations of glutathion reported by our group [10]. It is well known that the activation of *Nrf2* leads to the upregulation of proteins involved in the synthesis of glutathione, the main intracellular small molecule antioxidant [49].

Ramsey et al. (2007) [55] noted that surviving dopaminergic neurons in the brains of parkinsonian patients showed upregulation in *Nrf2* gene expression. This finding suggested that these neurons were capable of delay death and ultimately the neurodegenerative process. However, this group of authors points out that the protection mediated by *Nrf2* could be insufficient in neurons that have been lost [55].

Taking into account that *Nrf2* is an important transcription factor that regulates several *Nrf2*-target genes, such as HO-1 and NQO1, would be very interesting to evaluate the nigral gene expression of these and other genes related to *Nrf2* in the context of PPN neurotoxic lesion.

On the other hand, it is possible that the upregulation in nigral *Nrf2* gene expression could contribute to avoiding neuronal loss, although other morphological studies would be necessary to elucidate this aspect. Future studies aimed at detecting neurons immunopositive to tyrosine hydroxylase will contribute to deepen the survival of these cells 30 days after PPN lesion. Our results show similar nigral neuronal densities in the three time windows studied, following the PPN neurotoxic injury. However, it is very interesting that 30 days (4 weeks) after the PPN injury, the nigral neurons show a smaller surface area. Other authors have published that the injury of the PPN using a toxin selective for rat cholinergic neurons (Dtx-UII) produces a decrease in the density of TH+ dopaminergic neurons in the substantia nigra, seven weeks after the injury [3]. The smallest neuronal surface area observed in our work could be considered a sign of early atrophy that, 30 days after the pontine injury, still has no translation in the number of cells and therefore in cell density. The literature recognizes that the neurodegeneration process begins with a group of earlier events, modifying cell morphology and development, metabolism, and axonal transport, which progressively lead to a massive cell death rate. The brain often counterbalances those premature features with compensatory mechanisms at different levels—molecular and cellular [56].

## 5. Conclusions

The present work is a further attempt to mimic the slow and progressive course of nigral degeneration from a more vulnerable condition following neurotoxic injury of PPN. Our results indicate that the loss of nigral oxidative homeostasis is confluent with the increase in the nigral gene expression of *Nrf2*. This finding reinforces the criterion that the transcription factor *Nrf2* plays a key role in the adaptive response to oxidative stress. On the other hand, the results of our behavioral study underline the important role of PPN in both interhemispheric communication and motor control. The last aspect is very evident in the execution of tasks that challenge balance and posture control mechanisms on a raised and discontinuous surface.

## Figures and Tables

**Table 1:** Comparison of the time delayed for the rats before the first fault during the execution of Grid Test seven days after pedunculopontine nucleus lesion. One-way ANOVA test. The data represent x ± E.E.M.

Experimental Groups				
Variable	Non-Treated Rats	PPN Lesioned Rats	Sham-Operated Rats	Statistic
Time delayed before the first fault	11.90 ± 3.26	5.01 ± 1.10	11.37 ± 3.13	(F_(2, 35)_ = 2.10 *p* > 0.05)

Pedunculopontine nucleus: (PPN).

**Table 2:** Comparison of the time delayed for the rats before the first fault during the execution of grid test 30 days after pedunculopontine nucleus lesion ((F_(2,30)_ = 1.70 *p* > 0.05)). One-way ANOVA test. The data represent x ± E.E.M.

Experimental Groups				
Variable	Non-Treated Rats	PPN Lesioned Rats	Sham-Operated Rats	Statistic
Time delayed before the first fault	17.45 ± 5.26	7.34 ± 2.05	14.45 ± 4.44	(F_(2,30)_ = 1.70 *p* > 0.05)

Pedunculopontine nucleus: (PPN).
